# Ultrahigh Dose Rate Irradiation Regulates Mitochondrial DNA‐induced Interferon‐β Secretion via Cytochrome c Leakage

**DOI:** 10.1002/mco2.70457

**Published:** 2025-10-30

**Authors:** Jianfeng Lv, Jianhan Sun, Yunbin Luo, Juntao Liu, Di Wu, Yiyu Fang, Gerard Mourou, Senlin Huang, Gen Yang, Xueqing Yan

**Affiliations:** ^1^ State Key Laboratory of Nuclear Physics and Technology Peking University Beijing China; ^2^ Beijing Laser Acceleration Innovation Center Beijing China

1

Dear Editor,

Ultrahigh dose rate FLASH radiotherapy (FLASH‐RT), characterized by the ultrahigh dose rates (>40 Gy/s) and ultrashort treatment times (<1 s), represents a cutting‐edge technology in the field of radiotherapy during the last decade. FLASH‐RT has been demonstrated to spare normal tissues while maintaining equivalent tumor growth inhibition compared to conventional radiotherapy that delivers the same dose over several minutes [1], known as the FLASH effect. Nevertheless, mechanisms underlying the FLASH effect are still the subject of debate within the scientific community, and the hypotheses regarding radiolytic oxygen depletion and reactive oxygen species (ROS) reduction under FLASH irradiation have been extensively discussed [2]. As the primary organelles consuming oxygen and producing ROS within cells, mitochondria have been hypothesized to play a role in the efficacy of FLASH‐RT. Ionizing radiations cause excessive oxidative stress in mitochondria and facilitate cytochrome c and mitochondrial DNA (mtDNA) leakage into cytosol, provoking cellular apoptosis via caspase cascade and type‐I interferon‐related inflammation via cyclic‐GMP‐AMP synthase‐stimulator of the interferon gene (cGAS‐STING) pathway, respectively. The antagonism between cytosolic cytochrome c and mtDNA in cellular fate and immune response has been shown to be crucial to the radiotherapy efficacy [3], while the potential influence of irradiation dose rate on regulating these two pathways has not been studied yet, indicating a knowledge gap. This study aims to explore how alterations in irradiation dose rate regulate the apoptotic and inflammatory pathways associated with mitochondrial dysfunction.​

Cytochrome c leakage from mitochondria serves as the initiator of the caspase cascade and intrinsic apoptosis. In non‐tumorigenic human breast epithelial cells MCF‐10A, FLASH electron irradiation (61 Gy/s or 610 Gy/s) results in increasing cleaved caspase‐9 and cytochrome c leakage (characterized by weaker colocalization between cytochrome c and complex Vα) compared to the 0.36 Gy/s irradiation group (Figure 1A), and cytochrome c in the FLASH irradiation group exhibits a diffuse distribution throughout the cytosol. In contrast, FLASH irradiation mitigates both the caspase activation and cytochrome c leakage in human breast carcinoma cells MDA‐MB‐231 (Figure 1B), which keeps a high colocalization coefficient between cytochrome c and complex Vα. Besides, the complex Vα staining images show that cells subjected to electron irradiation exhibit significant mitochondrial network enlargement and morphological alteration compared to those in the control group.

**FIGURE 1:**
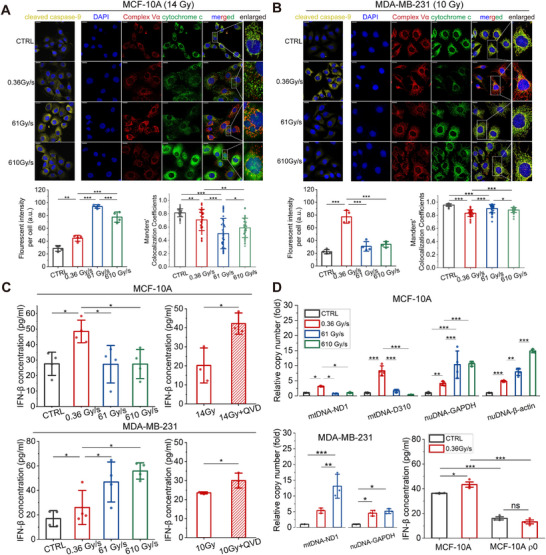
Mitochondria‐dependent responses to electron irradiation in MCF‐10A and MDA‐MB‐231 cells. (A) and (B) Representative immunofluorescence images of cleaved caspase‐9, complex V α subunit (complex Vα), and cytochrome c 9 h after irradiation in MCF‐10A (14 Gy) and MDA‐MB‐231 cells (10 Gy), respectively. Cells in the control group (CTRL) are sham irradiated. Scale bars: 20 µm. The corresponding mean fluorescent intensity of cleaved caspase‐9 per cell (*n* = 4) and colocalization analysis of complex Vα and cytochrome c (*n* = 4, 10 fields for each sample) are shown below the immunofluorescence images. (C) Supernatant interferon β (IFN‐β) concentration 48 h after irradiation and Q‐VD‐Oph (QVD) treatments in two cell lines (*n* = 4). (D) Cytosolic fraction of mitochondrial dsDNA (mtDNA) and nuclear dsDNA (nuDNA) 48 h after irradiation in two cell lines (*n ≥* 3), and the supernatant IFN‐β concentration in mtDNA‐depleted MCF‐10A cells (MCF‐10A ρ0) (*n* = 3). Data are presented as mean ± SD. *p < 0.05, **p < 0.01, and ***p < 0.001.

Previous studies have demonstrated that apoptotic caspases facilitate suppression of type I interferons production [4]. This study quantifies interferon‐β (IFN‐β) concentration in cell culture supernatants at 48 h post‐irradiation to further investigate this regulatory mechanism. Intriguingly, FLASH irradiation reduces the IFN‐β secretion in MCF‐10A cells but enhances that in MDA‐MB‐231 cells (Figure 1C), and these changes are opposite to those in caspase activation and cytochrome c leakage. Furthermore, for both cell lines, cells treated with 0.36 Gy/s irradiation and caspase inhibition (Q‐VD‐oph) secrete more IFN‐β compared to the irradiated cells without a caspase inhibitor (Figure 1C). These results unveil that IFN‐β secretion can be suppressed by irradiation‐induced caspase activation, whereas weaker cytochrome c leakage and caspase activation promote the IFN‐β secretion.

The upstream signaling pathway regulating IFN‐β production is also of interest. Shi et al. [5] reported the reduced cytosolic dsDNA and cGAS‐STING pathway activation in the mouse intestine post X‐ray FLASH‐RT, leading to reduced type‐I interferon‐associated inflammation and T‐cells recruitment. To explore the contribution of mtDNA to IFN‐β secretion in this study, specific primers targeting mtDNA and nuclear DNA (nuDNA) are designed to assess the relative copy number of cytosolic dsDNA using real‐time quantitative PCR assays (Figure 1D). For MCF‐10A cells, the cytosolic mtDNA accumulation after FLASH irradiation (61 Gy/s) is significantly lower than that in the 0.36 Gy/s irradiation group, while such a difference is not observed for cytosolic nuDNA in three independent assays. In contrast to non‐tumorigenic cells, FLASH irradiation enhances the cytosolic mtDNA accumulation in MDA‐MB‐231 carcinoma cells. Although an increasing trend in cytosolic nuDNA accumulation is observed following FLASH irradiation, the statistical significance remains unconfirmed. The consistent changes observed in cytosolic mtDNA accumulation and IFN‐β concentration are likely attributable to the mitochondrial dysfunction and caspase activation. The ρ0 cells, characterized by mtDNA depletion, are utilized to elucidate the functional role of mtDNA in eliciting IFN‐β secretion. The irradiated and non‐irradiated MCF‐10A ρ0 cells show no difference in IFN‐β concentration, and the supernatant IFN‐β concentrations of MCF‐10A ρ0 cells are much lower than those of MCF‐10A cells, indicating the deficient ability of stimulating IFN‐β production in the absence of mtDNA. Such results in MDA‐MB‐231 ρ0 cells are also obtained. Given the pivotal role of interferons in immune regulation, their altered expression at the cellular level may modulate immunogenic cell death and immune activation within irradiated tissues.

Our observations regarding cytochrome c suggest a fundamental physicochemical process underlying mitochondrial dysfunction, initiated by the disruption of electron transport chain function and the subsequent detachment of cytochrome c after exposure to FLASH irradiation. The resulting change in oxidative phosphorylation may derive differential metabolic reprogramming between normal and carcinoma cells, given that some carcinoma cells primarily rely on aerobic glycolysis rather than mitochondrial oxidative phosphorylation for energy production. The electron transport chain and cellular metabolism could serve as intriguing targets to explore systematic mechanisms of the FLASH effect.

Overall, we demonstrate that FLASH irradiation reduces IFN‐β secretion in non‐tumorigenic MCF‐10A cells by enhancing the cytochrome c leakage and caspase activation, while it can increase IFN‐β secretion in MDA‐MB‐231 carcinoma cells by suppressing the apoptotic caspases. Notably, IFN‐β secretion exhibits dependency on the cytosolic mtDNA accumulation. The marked difference in cytochrome c leakage between carcinoma and normal cells following FLASH irradiation further supports the involvement of mitochondria‐mediated mechanisms, which appear to regulate both apoptotic signaling and inflammatory responses in FLASH radiobiology.

## Author Contributions

**Jianfeng Lv**: methodology, formal analysis, investigation, data curation, visualization, and writing‐original draft. **Jianhan Sun** and **Yunbin Luo**: methodology, formal analysis, investigation, data curation, validation, and visualization. **Juntao Liu**, **Di Wu**, and **Yiyu Fang**: methodology, investigation. **Gerard Mourou**: methodology and conceptualization. **Senlin Huang**, **Gen Yang**, and **Xueqing Yan**: supervision, conceptualization, resources, writing‐review, and editing. All authors reviewed the manuscript.

## Funding

This work was supported by grants from the National Key Research and Development Program of China (Nos. 2019YFF01014400, 2023YFC2413200, and 2023YFC2413201), National Natural Science Foundation of China (Nos. 12375334 and 11921006), and International Collaboration Fund for Creative Research Teams (ICFCRT) of NSFC (W2541004).

## Ethics Statement

The authors have nothing to report.

## Conflicts of Interest

The authors declare no conflicts of interest.

## Supporting information




**Supporting File 1**: mco270457‐sup‐0001‐SuppMat.docx

## Data Availability

Data that support the findings of this study have been deposited in FigShare at https://doi.org/10.6084/m9.figshare.25957660.v1.
