# Supplementary material for: Ultrahigh Dose Rate Irradiation Regulates Mitochondrial DNA‐induced Interferon‐β Secretion via Cytochrome c Leakage
Source: MedComm (2020). 2025 Oct 30;6(11):e70457. doi: 10.1002/mco2.70457 (PMC12572942; doi:10.1002/mco2.70457)
Supplement: Supplementary file 1 — Supporting File 1: mco270457‐sup‐0001‐SuppMat.docx [file MCO2-6-e70457-s001.docx]

Supplementary Materials for

**Ultrahigh Dose Rate Irradiation Regulates mtDNA-Induced Interferon-β Secretion via Cytochrome c Leakage**

Jianfeng Lv^1,2^, Jianhan Sun^1^, Yunbin Luo^1,2^, Juntao Liu^1^, Di Wu^1,2^, Yiyu Fang^1,2^, Gerard Mourou^1,2^, Senlin Huang^1*^, Gen Yang^1,2*^, Xueqing Yan^1,2*^.

^1^State Key Laboratory of Nuclear Physics and Technology, Peking University, Beijing, 100871, China

^2^Beijing Laser Acceleration Innovation Center, Beijing, 101407, China

^*^Correspondence: Senlin Huang: [huangsl@pku.edu.cn](mailto:huangsl@pku.edu.cn); Gen Yang: [gen.yang@pku.edu.cn](mailto:gen.yang@pku.edu.cn); and Xueqing Yan: [x.yan@pku.edu.cn](mailto:x.yan@pku.edu.cn)

**Materials and Methods**

**Cell culture**

Non-tumorigenic human breast epithelial cells MCF-10A (ATCC CRL-10317) and human breast carcinoma cells MDA-MB-231 (ATCC HTB-26) were obtained from American Type Culture Collection (ATCC) for comparative analysis. Cells are authenticated through STR profiling and tested negative for mycoplasma contamination. MCF-10A cells were grown in DMEM/F12 (Pricella) containing 5% HS, 20ng/mL EGF, 0.5μg/mL Hydrocortisone, 10μg/mL Insulin, 1% non-essential amino acids and 1% penicillin/streptomycin (P/S). MDA-MB-231 cells were cultured in Dulbecco's Modified Eagle Medium (DMEM) supplemented with 10% FBS and 1% P/S. To prepare the mtDNA depleted cells (ρ0 cells), the culture medium was further supplemented with 50 ng/ml ethidium bromide, 1mM sodium pyruvate and 50 μg/ml uridine, and the cells were incubated for 5 passages. Quantitative real-time PCR was used to confirm the mtDNA depletion. All the cells were incubated at 37 ℃ with 5% CO_2_ and 21% O_2_.

**Ultra-high dose rate electron irradiation**

The direct current-superconducting radiofrequency (DC-SRF) photocathode electron gun can produce stable electron beams with the energy of 1.79 MeV. The repetition rate of electron micro-pulses is determined by the driving laser, which can work at two modes of 1 MHz and 81.5 MHz. The micro-pulse duration (picosecond to sub-picosecond) and charge per micro-pulse (picocoulomb) can also be continuously adjusted by the driving laser. Based on this photocathode electron gun, a specialized beam line utilizing a scatterer and collimator was applied for delivering electrons to samples with uniform dose distribution and controllable dose rates (10^-1^ ~10^4^ Gy/s). Diameter of the round irradiation field is 35 mm.

**Clonogenic cell survival assay**

Cobalt-60 γ-ray was used to assess these cell lines’ radio-sensitivity used in this study. Cells for each group were seeded in the 6-well plates one day before the irradiation. Irradiation groups were irradiated under the dose rate of 0.36 Gy/s, and the control groups were blocked with lead bricks. The linear energy transfer (LET) of Cobalt-60 γ-ray in cells is about 0.2 keV/μm. After irradiation, cells were cultured for 10 days and the culture media were replaced with the fresh every 3 days. Then the adherent cells were fixed with 4% paraformaldehyde for 10 minutes and dyed with 0.5% crystal violet. The colonies whose diameters were larger than 0.3 mm were counted to calculate the survival fraction (SF). Doses used in this study (MCF-10A: 14 Gy; MDA-MB-231: 10 Gy) correspond to 5‰ cell survival fraction based on clonogenic cell survival assay. We selected these high doses based on previous literatures in order to enhance the potential differences caused by FLASH irradiation.

**Dose monitoring and calculation**

The electron energy (1.79 MeV) was measured by a bending magnet in the beamline, and other beam parameters were monitored by a fast-current-transformer and oscilloscope. Radiochromic films (RCF, EBT-3 type) were placed in front of the cell samples for dose measurement. An Epson Perfection V700 scanner was used to scan the irradiated RCF in the transmission mode, and the calibration of RCF dose response to ionization radiations was referenced to the previous literatures^1,2^. The dose delivery to cells differed from that measured by RCF due to the electron energy decrease from RCF to cells. The ratio of the doses in cells and RCF is 1.25 as calculated by Monte Carlo simulations with Geant4^3-5^.

**Immunofluorescence**

For immunofluorescence observation, 4×10^4^ cells were seeded to the confocal petri dish with 15 mm glass bottom (NEST, no. 801002) one day before irradiation. At the indicated time post irradiation (9 hours for cytochrome c, complex Ⅴα, and cleaved caspase-9 observation), cells were fixed with 4% paraformaldehyde for 10 minutes for storage or immediate use. Immunofluorescence processes were referenced to the instructions of antibody products. Briefly, samples were permeabilized with 0.1% Triton X-100 for 10 minutes at 4 ℃, and then were blocked with blocking buffer for 45 minutes at room temperature before incubating with the primary antibodies (anti-cytochrome c and anti-complex Ⅴα: Abcam, no. Ab110417; anti-cleaved caspase-9: Affinity, no. AF5244) at 4 ℃ overnight. In the following day, samples were further incubated with corresponding secondary antibodies for 1 hour at room temperature. Finally, the samples were mounted with mounting medium containing DAPI (Beyotime, no. P0131). For each imaging field, *z*-stack fluorescence images with a spacing of 1 μm in the *z*-direction were acquired by using an LSM-700 confocal microscope (Zeiss). The function of “extended depth of focus” in the software Zen was applied to process the *z*-stack and generate a single composite image, which was then quantitatively analyzed with ImageJ or demonstrated as the representative image. For each sample, more than 10 imaging fields were randomly selected, and more than 300 cells were analyzed for each group.

**Cytosolic dsDNA extraction and Real-time quantitative PCR**

48 hours post irradiation, total DNA and cytosolic DNA were extracted and quantified as previously described^6-8^. Briefly, cells collected from 6-well plate were divided into two equal aliquots. The first aliquot was suspended in 500 µL of 50 mM NaOH and boiled for 30 min to dissolve the DNA. Then, 50 µL of 1 M tris-HCl (pH 8.0) was added to balance the pH and centrifuged at 17,000 g for 10 min to separate cellular debris. These extracts were used to quantify total dsDNA. The second aliquot was suspended in 500 µL of buffer with 150 mM NaCl, 50 mM Hepes (pH 7.4), and digitonin (25 μg/ml; HARVEYBIO, no. LS1463). The mix were then incubated end-over-end for 10 min on ice to enable selective membrane permeabilization and centrifuged at 980 g for 3 minutes 3 times to pellet intact cells. Finally, the cytosolic supernatants were transferred to new tubes and spun at 17,000 g for 10 min to pellet any leftover cellular debris. These extracts were used to quantify cytosolic dsDNA. All dsDNA samples were purified with DNA Clean & Concentrator-5 (ZYMO RESEARCH, no. D4013) before further use. To detect the differences of nuDNA and mtDNA leakage to cytosol after irradiations with different dose rates, the real-time quantitative PCR (qPCR) was performed on 7500 FAST real-time PCR system with Tag Pro Universal SYBR Master Mix (Vazyme, no. Q712-02) according to the instruction manuals. Ct values obtained from whole-cell extracts served as normalization controls for the Ct values from cytosolic extracts. The nuDNA and mtDNA primers used in qPCR are listed below. nuDNA β-actin(F): ACCCACACTGTGCCCATCTAC; nuDNA β-actin(R): TCGGTGAGGATCTTCATGAGGTA; nuDNA GAPDH(F): AGCCACATCGCTCAGACACCA; nuDNA GAPDH(R): GCAAATGAGCCCCAGCCTTC; mtDNA ND1 (F): CACCCAAGAACAGGGTTTGT; mtDNA ND1 (R): TGGCCATGGGTATGTTGTTAA; mtDNA D310 (F): CACAGACATCATAACAAAAAATTTCC; mtDNA D310 (R): GGTGTTAGGGTTCTTTGTTTTTGG.

**Interferon-β detection**

After irradiation, culture medium was immediately replaced with 1 ml the fresh for each well that contains about 10^6^ adherent cells. For the cells with caspase inhibition, the fresh culture medium is further supplemented with Q-VD-OPh (Aladdin, no. Q275003) (2 μM concentration). Then the media supernatants were transferred to fresh tubes after 48 hours incubation. These samples were centrifuged at 1000 g for 20 minutes for immediate use or storage at -20 ℃. The human IFN-β ELISA kit was used to quantify the IFN-β concentrations.

**Statistical analysis**

Origin (version 2022) (OriginLab Corp., Northampton, MA, USA.) was used for statistical analysis and graph generation. Each experiment was repeated with at least 3 biologically independent samples. Comparison between two groups was performed using unpaired *t*-test. Comparison between outcomes of different dose rates was performed using one-way analysis of variance (ANOVA) with Holm-Bonferroni's multiple comparisons test. All the quantitative results were presented as mean±SD. For all graphs, *p<0.05; **p<0.01; and ***p<0.001.

1. Reinhardt S, Hillbrand M, Wilkens JJ, Assmann W. Comparison of Gafchromic EBT2 and EBT3 films for clinical photon and proton beams. *Med Phys*. Aug 2012;39(8):5257-5262.

2. Campajola L, Casolaro P, Di Capua F. Absolute dose calibration of EBT3 Gafchromic films. *J Instrum*. Aug 2017;12:P08015.

3. Agostinelli S, Allison J, Amako K, et al. GEANT4-a simulation toolkit. *Nucl Instrum Meth A*. Jul 1 2003;506(3):250-303.

4. Allison J, Amako K, Apostolakis J, et al. Geant4 developments and applications. *Ieee T Nucl Sci*. Feb 2006;53(1):270-278.

5. Allison J, Amako K, Apostolakis J, et al. Recent developments in GEANT4. *Nucl Instrum Meth A*. Nov 1 2016;835:186-225.

6. Yang YQ, Wu M, Cao DQ, et al. ZBP1-MLKL necroptotic signaling potentiates radiation-induced antitumor immunity via intratumoral STING pathway activation. *Sci Adv*. Oct 2021;7(41):eabf6290.

7. Hu MJ, Zhou M, Bao XH, et al. ATM inhibition enhances cancer immunotherapy by promoting mtDNA leakage and cGAS/STING activation. *J Clin Invest*. Feb 1 2021;131(3):e139333.

8. Li WW, Lu L, Lu JJ, et al. cGAS-STING-mediated DNA sensing maintains CD8(+) T cell stemness and promotes antitumor T cell therapy. *Sci Transl Med*. Jun 24 2020;12(549)
